# Pharyngeal pumping in *Caenorhabditis elegans* depends on tonic and phasic signaling from the nervous system

**DOI:** 10.1038/srep22940

**Published:** 2016-03-15

**Authors:** Nicholas F. Trojanowski, David M. Raizen, Christopher Fang-Yen

**Affiliations:** 1 Department of Neurology, Perelman School of Medicine, University of Pennsylvania, Philadelphia, 19104 PA, USA; 2 Department of Bioengineering, School of Engineering and Applied Science, University of Pennsylvania, Philadelphia, 19104 PA, USA; 3 Department of Neuroscience, Perelman School of Medicine, University of Pennsylvania, Philadelphia, 19104 PA, USA.

## Abstract

Rhythmic movements are ubiquitous in animal locomotion, feeding, and circulatory systems. In some systems, the muscle itself generates rhythmic contractions. In others, rhythms are generated by the nervous system or by interactions between the nervous system and muscles. In the nematode *Caenorhabditis elegans,* feeding occurs via rhythmic contractions (pumping) of the pharynx, a neuromuscular feeding organ. Here, we use pharmacology, optogenetics, genetics, and electrophysiology to investigate the roles of the nervous system and muscle in generating pharyngeal pumping. Hyperpolarization of the nervous system using a histamine-gated chloride channel abolishes pumping, and optogenetic stimulation of pharyngeal muscle in these animals causes abnormal contractions, demonstrating that normal pumping requires nervous system function. In mutants that pump slowly due to defective nervous system function, tonic muscle stimulation causes rapid pumping, suggesting tonic neurotransmitter release may regulate pumping. However, tonic cholinergic motor neuron stimulation, but not tonic muscle stimulation, triggers pumps that electrophysiologically resemble typical rapid pumps. This suggests that pharyngeal cholinergic motor neurons are normally rhythmically, and not tonically active. These results demonstrate that the pharynx generates a myogenic rhythm in the presence of tonically released acetylcholine, and suggest that the pharyngeal nervous system entrains contraction rate and timing through phasic neurotransmitter release.

Rhythmic muscle contractions are required for many aspects of physiology and behavior, from circulation to locomotion^1^. These rhythms can be described as myogenic, if intrinsic oscillations of membrane currents in the muscles drive contractions, or neurogenic, if a network of neurons acts as a central pattern generator (CPG) to drive muscle contraction. For example, vertebrate heart muscle generates its own rhythms. The autonomic nervous system modulates the rate and strength of cardiac contraction but does not provide any beat-to-beat timing information^2^, and innervation of the heart is dispensable for coordinated and effective cardiac function^3^. In contrast, a neural circuit in the vertebrate spinal cord controls locomotion by acting as a neural pacemaker, producing patterned activity that drives contraction of passively responding skeletal muscles^4^.

Myogenic and neurogenic rhythms are not mutually exclusive: in some systems both the nervous system and muscles are capable of generating rhythms independently, and interact to generate rhythmic behavior. For example, leech heart motor neurons display rhythmic activity and entrain the myogenic rhythmic contractions of the heart^5^. Similarly, the crustacean pyloric dilator muscle exhibits a myogenic rhythm that is entrained by rhythmic activity in the stomatogastric neural system^6^. By contrast, mollusc heart motor neurons modulate heart rate over long time scales without entraining the heartbeat^7^.

The mechanisms that underlie rhythmic behaviors have been most studied in invertebrates such as leeches, crustaceans, and molluscs due to the small number of neurons and relative ease of electrophysiological recordings in these animals. The ability to electrophysiologically identify the functional synaptic connectivity between neurons in these systems has enabled researchers to determine the roles of intrinsic and synaptic properties of individual neurons and muscles in generating rhythmic behaviors^1^,^8^,^9^. However, these organisms are not amenable to genetic approaches, limiting their utility for investigation of the genetic and molecular bases of rhythm generation in nervous systems and muscles.

The nematode *Caenorhabditis elegans* represents a unique and powerful model for elucidating the genetic, neural, and muscular bases of behavior^10^,^11^. Among its strengths are its compact, extraordinarily well-mapped nervous system^12^,^13^, genetic manipulability, and optical transparency. The development of optical^14^,^15^ and electrophysiological^16^ methods for manipulating and monitoring neural activity has begun to enable analysis of the physiology and functional connectivity of *C. elegans* neural circuits. Such investigations apply a conceptual approach similar to that developed in leeches, crustaceans, and gastropods while leveraging the extensive genetic toolkit available in worms. Thus, *C. elegans* is well suited to provide insights into mechanisms that underlie rhythmic behaviors.

C. *elegans* feeds on bacterial food via rhythmic contractions and relaxations of its pharynx (Fig. 1), a neuromuscular pump with similarities to the vertebrate and invertebrate heart. Like these hearts, the pharynx is a tube of electrically coupled muscle cells^17^,^18^,^19^,^20^ that pumps throughout the life of the animal. It responds to a variety of neurotransmitters and neuromodulators^21^, and relies on T- and L-type Ca^2+^ channels for its action potential^22^,^23^. The pharynx possesses a nervous system, a network of 20 neurons of 14 types, that is largely independent of the extra-pharyngeal nervous system and accounts for all chemical synapses onto pharyngeal muscle^13^.

The role of the pharyngeal nervous system in the generation of rhythmic pharyngeal behavior is not yet clear. Laser ablation of all pharyngeal neurons does not completely abolish pharyngeal pumping^24^, nor does optogenetic hyperpolarization of all cholinergic pharyngeal motor neurons^25^,^26^, which normally excite pumping^24^,^25^,^27^. On the basis of these findings, the pharyngeal pumping rhythm has been described as myogenic^24^,^28^. However, pumping is abolished by genetic manipulations that eliminate cholinergic synaptic transmission^29^,^30^ or all synaptic transmission^31^,^32^,^33^, indicating that some nervous system function is required for pumping.

Of the 20 pharyngeal neurons, the two cholinergic MC motor neurons appear to be the most important for regulation of rapid pumping: MC ablation dramatically decreases pump rate^24^,^27^, and optogenetic stimulation or inhibition of the MC neurons increases or decreases pump rate, respectively^25^. The MC neurons are activated by serotonin (5-HT)^34^ and appear to act primarily via a nicotinic acetylcholine (ACh) receptor containing the non-α subunit EAT-2, as *eat-2* mutants resemble MC-ablated animals^25^,^27^,^35^,^36^. Electropharyngeograms (EPGs), extracellular recordings of pharyngeal muscle electrical activity^37^, reveal a very brief MC- and EAT-2-dependent depolarization preceding each muscle action potential during rapid pumping^27^. This depolarization may represent a response to pulsed neurotransmitter release from the MC neurons, but this idea is challenging to test since the activity patterns of the MC neurons are unknown and currently difficult to measure.

We explored how the nervous system and pharyngeal muscle interact to control pumping, with the goal of comparing the mechanisms of pharyngeal contraction generation with those found in vertebrate and invertebrate hearts and other rhythmic systems. Our results demonstrate that the pharyngeal muscle generates a myogenic rhythm only in the presence of tonically released ACh, and suggest that the MC neurons stimulate pumping by rhythmically exciting and entraining the pharyngeal muscle rhythm in a manner similar to that by which the leech heartbeat is controlled by heart motor neurons.

## Results

### Pharyngeal pumping acutely requires nervous system function

The finding that pharyngeal pumping persists after laser ablation of the entire pharyngeal nervous system^24^ or after hyperpolarization of excitatory pharyngeal cholinergic neurons^25^,^26^, yet is abolished in mutants lacking ACh release^29^,^30^,^31^,^32^,^33^ suggests that ACh from the extra-pharyngeal nervous system is sufficient to induce feeding. However, since severe synaptic transmission mutations cause chronic changes in animal physiology and development, it is possible that the lack of feeding observed in these mutants may be explained by developmental abnormalities.

To test the role of the nervous system in pumping while avoiding the confounding issue of abnormal development in mutant backgrounds, we sought to determine if the nervous system is acutely required for pumping. In order to acutely silence the nervous system, we expressed a histamine-gated chloride channel (HisCl) in all neurons using the *Ptag-168* promoter^38^. HisCl activation has been shown to silence neurons in every case tested, both in *C. elegans*^38^,^39^ and in *Drosophila*^40^. Therefore, in worms expressing pan-neuronal HisCl, exogenous histamine is expected to lead to hyperpolarization of both pharyngeal and extra-pharyngeal neurons^38^, including excitatory cholinergic pharyngeal neurons such as the MCs. After 15 minutes on a 2% agarose pad containing 10 mM histamine, pumping completely ceased in worms expressing pan-neuronal HisCl (n > 80), while pumping persists on 2% agarose pads in worms lacking pan-neuronal HisCl^41^. Exposure of these worms to histamine does not dramatically affect pumping, as worms raised on histamine do not show severe growth defects^38^,^39^. Therefore, unlike the vertebrate, leech, and mollusc hearts, the pharynx requires a signal from the nervous system to produce myogenic contractions. Since ablation of the pharyngeal nervous system does not abolish pumping^24^, it appears that this signal can come from the extra-pharyngeal nervous system. However, since extra-pharyngeal neurons do not synapse on pharyngeal muscle, they cannot provide pump-to-pump timing information.

### Normal pharyngeal muscle coordination requires the nervous system

**Figure 1:**
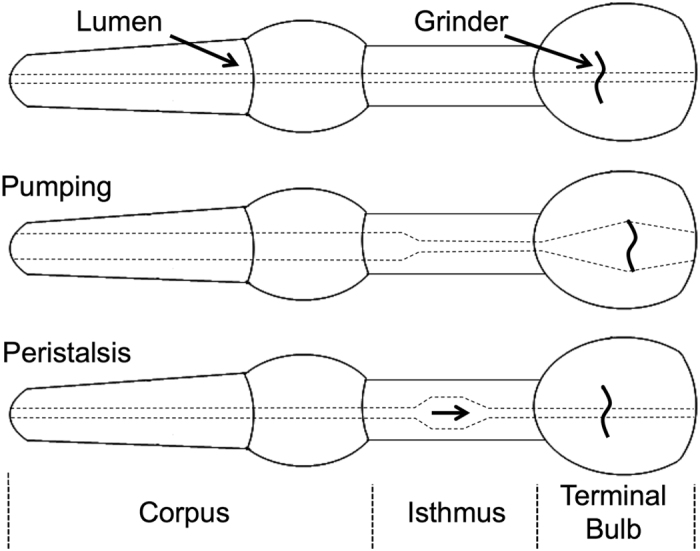
The pharynx consists of three functional units, the corpus, isthmus, and terminal bulb. During a pump, food enters via the corpus. It is then transferred along the isthmus via posteriorly propagating peristaltic waves before being broken up by the cuticular grinder in the terminal bulb during the subsequent pump. Anterior is left.

We sought to better understand the role of the pharyngeal nervous system in coordinating pharyngeal pumps. The pharynx can be divided into three functional units^13^ (Fig. 1). The anterior end of the pharynx contains the corpus, which draws in the bacterial food during muscle contraction. The posterior end of the pharynx contains the terminal bulb, which houses the grinder, three cuticular plates that crush the bacteria so their contents can be absorbed by the intestine. The isthmus connects the corpus and terminal bulb. Pharyngeal muscle fibers are oriented radially, so muscle contraction opens the lumen and relaxation closes it. During pumping, it is necessary for different parts of the pharynx to contract with slightly different timing to effectively transport food to the intestine^42^. A pharyngeal pump begins with the nearly simultaneous contraction of the corpus and the terminal bulb, drawing food particles into the pharyngeal lumen, followed by contraction of the anterior isthmus. After approximately 200 ms, these muscles begin to relax. The anterior tip of the corpus relaxes first, preventing food particles from escaping when the rest of the muscles relax^43^. Likewise, the corpus relaxes before the isthmus, allowing bacteria to be trapped in the anterior isthmus^43^,^44^. Posteriorly-propagating contractions of the posterior isthmus, known as peristalsis, transport bacteria from the anterior isthmus to the terminal bulb after about one out of every four pumps^45^.

To gain insight into which aspects of pharyngeal pumping require the nervous system, we sought to determine the extent to which direct stimulation of pharyngeal muscle in the absence of nervous system function recapitulates normal muscle contraction patterns. In the absence of neural input, the vertebrate heart generates motions that are essentially the same as those observed with neural input; neural input modulates only the rate and force of cardiac contractions^2^. In contrast, in the leech heart, the electrical activity of the muscle is altered when the nervous system is hyperpolarized^5^. To test whether the pharynx produces motions in the absence of neural input that are similar to those produced in the presence of neural input, we silenced the nervous system using pan-neuronal HisCl activation and then stimulated the muscle directly using the light-activated excitatory opsin Chrimson expressed in pharyngeal muscle^46^. We used high-speed video recordings to examine the muscle contraction patterns of these worms in response to 200 ms optogenetic stimulation of pharyngeal muscle in the presence of histamine.

**Figure 2:**
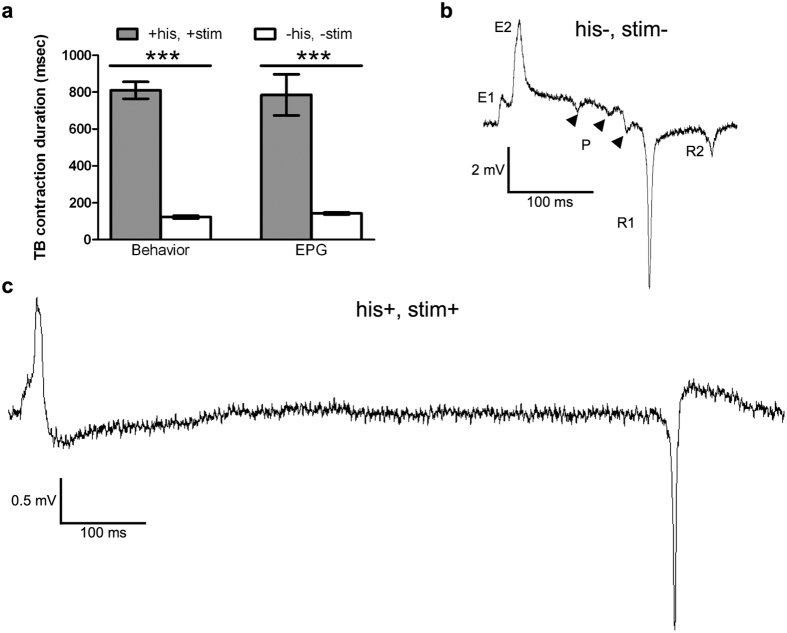
The nervous system modulates terminal bulb contraction rate. (**a**) 200 ms optogenetic stimulation of pharyngeal muscle in worms expressing pan-neuronal HisCl and in the presence of histamine caused pumps with long terminal bulb contractions as measured by either high-speed video recordings of muscle contractions (behavior) or electrophysiological recordings (EPG). For video analysis, N = 15 worms for light−, his−, N = 37 worms for light+, his+. For EPG analysis, N = 11 for light−, his−, N = 15 for light+, his+. Statistical significance was calculated using a two-tailed Student’s *t*-test. ***p < 0.001. (**b**) Example EPG trace for his-, light- worm. The various components of the EPG are labeled. Arrowheads indicate P spikes. (**c**) Example EPG trace for his+, light+ worm.

We compared pan-neuronally hyperpolarized animals with muscle optogenetic stimulation to animals of the same strain without histamine or muscle stimulation, and we found two striking differences. First, in 31/37 experimental worms, we observed contraction in the terminal bulb but not in the corpus in response to optogenetic stimulation. The remaining 6/37 experimental worms showed feeble corpus contractions. By contrast, in the absence of histamine and optogenetic muscle stimulation, all worms of this strain had normal contractions of the corpus in addition to the terminal bulb (N = 15). The difference between the experimental and control worms is statistically significant (p < 0.05, Z test). The other striking difference was that the duration of terminal bulb contraction in the presence of histamine and optogenetic muscle stimulation was 805 ± 52 ms, (mean ± SEM, Fig. 2a), far exceeding the 200 ms stimulus duration. These data suggest that pharyngeal muscle contractions are defective in the acute absence of nervous system function, demonstrating that the nervous system plays a role in setting contraction duration and pattern.

Three classes of mutants have been identified with increased pump duration^24^. Mutants defective in the neurotransmission of the M3 glutamatergic inhibitory motor neurons, including those lacking the vesicular glutamate transporter gene *eat-4*^47^ or the avermectin-sensitive glutamate-gated Cl^−^ channel gene *avr-15*^48^, have prolonged contractions due to lengthened pharyngeal action potentials. Prolonged contractions due to long actions potentials are also seen in mutants with increased pharyngeal excitability, including loss-of-function mutations in the Na^+^/K^+^ transporter α-subunit gene *eat-6*^49^ or the K^+^ channel gene *exp-2*^50^, or gain-of-function mutations in the L-type Ca^2+^ channel α1 subunit gene *egl-19*^26^,^47^. In contrast, worms with increased Gαq signaling due to mutations in the RGS protein *eat-16* or the Gβ_5_ subunit gene *gbp-2*, or overexpression of muscarinic ACh receptor gene *gar-3*, show muscle contractions that outlast pharyngeal action potentials^51^,^52^. Thus, multiple mechanisms could explain the long contractions observed when the nervous system is silenced and the muscle is optogenetically stimulated.

To differentiate between these mechanisms, we recorded EPGs. During a pharyngeal pump, the EPG shows two prominent features arising from muscle activity: an E2 spike that indicates muscle depolarization, and the R1 and R2 spikes that represent repolarization of the corpus and terminal bulb muscles, respectively (Fig. 2b). P spikes represent inhibitory postsynaptic potentials (IPSPs) evoked by the M3 neurons during the pump^37^,^42^,^48^. When we stimulated the pharyngeal muscle in worms in which the nervous system was silenced via the HisCl channel, we found that pharyngeal muscle action potential duration, measured as the time between the E2 and R1 spikes, was dramatically increased (Fig. 2a,c), suggesting that increased pump duration is due to either decreased M3 activity or altered pharyngeal excitability. However, while we noted that P spikes were absent in these worms, the prolongation of the pumps we observed was far more extreme than that found in animals lacking M3 neurotransmission^42^ or even in animals lacking all pharyngeal neurons^24^. This suggests that the nervous system promotes terminal bulb relaxation by altering the membrane or ion channel properties or Ca^2+^ signaling in the pharyngeal muscle.

### Tonic depolarization of pharyngeal muscle can stimulate rapid pumping in mutants with defective neurotransmission

Having established that normal myogenic pharyngeal muscle contraction requires nervous system activity but not precise timing information, we next sought to determine if the nervous system provides phasic input to regulate rapid pumping, as in the leech heartbeat, or modulatory input that regulates the myogenic contraction rate, as in vertebrate and mollusc hearts. Recent reports have shown that both phasic and tonic optogenetic excitation of pharyngeal muscle can stimulate rapid pumping^26^,^46^. However, the role of the nervous system in this rapid pumping is unclear.

**Figure 3:**
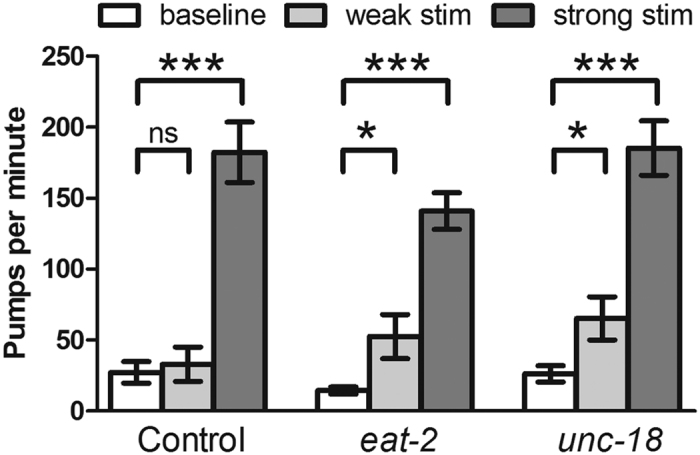
Tonic optogenetic muscle stimulation causes rapid pumping in mutants with defects in excitatory transmission. Strong optogenetic stimulation of pharyngeal muscle produced similar pumping rates in control worms, *eat-2* mutants, and *unc-18* mutants, while weak optogenetic stimulation excited pumping in *eat-2* and *unc-18* mutants but not control worms. For control worms, we selected worms with low basal pumping rates so the data would be more comparable to that of *eat-2* and *unc-18* mutants. N = 8–10 worms. Statistical significance was calculated using a two-tailed Student’s *t*-test. *p < 0.05, ***p < 0.001.

To test if rapid, phasic excitation from the MC or other neurons is essential for rapid pumping, we measured the pumping rate during tonic optogenetic stimulation of pharyngeal muscle in two mutants with defective neurotransmission. In worms with normal MC neurons and the EAT-2-containing receptor, EPG recordings in the presence of 5-HT reveal that each pharyngeal muscle action potential is preceded by a small depolarization resembling an excitatory postsynaptic potential (EPSP) (the E1 spike in Fig. 2b)^27^, and absence of MC or EAT-2 causes a dramatic decrease in pump rate and the disappearance of the E1 spike^24^,^35^. To test if MC function via the EAT-2-containing receptor is required for rapid pumping in the presence of 5-HT, we optogenetically stimulated the pharyngeal muscle in *eat-2* mutants. Whereas optogenetic stimulation of the MC neurons in these mutants in the presence of 5-HT caused only a small increase in pumping^25^, we found that tonic optogenetic pharyngeal muscle stimulation under similar conditions caused a dramatic increase in pump rate similar to that seen in control worms (Fig. 3), demonstrating that rapid pumping can occur in the absence of nicotinic MC neurotransmission.

Next, to test whether direct muscle stimulation can trigger rapid pumping even when neurotransmission is severely and globally impaired (but not abolished), we repeated this experiment in the strongest viable synaptic null mutant available, *unc-18*^53^. Null mutants for syntaxin (*unc-64*)^31^, SNAP-25 (*ric-4*)^54^, synaptobrevin (*snb-1*)^32^, and *unc-13*^33^ are not viable, indicating that complete absence of synaptic release is lethal. Thus, since *unc-18* null mutants are sick and slow growing but viable, we reasoned that these mutants have sufficient nervous system function to permit myogenic pumping, but not enough to stimulate rapid pumping, hence their decreased basal pump rate. A recent report found that pumping could be triggered by rhythmically depolarizing pharyngeal muscle in an *unc-13* partial loss of function mutant, though only to a rate similar to that of unstimulated *unc-13* mutants and at least five-fold slower than the rate observed in wild-type animals^26^.

As with *eat-2* mutants, optogenetic muscle stimulation in *unc-18* mutants caused rapid pumping at a rate similar to that of control worms (Fig. 3), much faster than that normally observed in these mutants^53^, demonstrating that rhythmic input from the nervous system is not required for rapid pumping. When subjected to a milder optogenetic stimulus, *eat-2* and *unc-18* mutants but not control worms showed an increase in pumping rate (Fig. 3), suggesting an activity-dependent homeostatic increase in membrane excitability may occur in these mutants^55^. This is supported by evidence that in *eat-2* mutants, the resting membrane potential is depolarized and unstable^56^. Together, these results show that tonic depolarization of pharyngeal muscle is sufficient to trigger rapid pumping even when the excitatory inputs to the pharynx are defective.

### Tonic stimulation of pharyngeal muscle and tonic stimulation of cholinergic neurons yield distinct electrical activity patterns

Both tonic and phasic stimulation of pharyngeal muscle are individually sufficient to drive rapid pumping at physiological rates^26^,^46^. Therefore, it is unclear whether MC activity during normal rapid pumping is rhythmic or tonic. On one hand, EPG recordings reveal EAT-2-dependent depolarizations in pharyngeal muscle preceding each muscle action potential during rapid pumping in the presence of 5-HT (the E1 spike in Fig. 2b). These spikes have been hypothesized to represent EPSPs from rhythmic MC action potentials, which entrain the pharyngeal muscles^27^. On the other hand, since these depolarizations are observed during rapid pumping but not slow pumping^27^, and rapid neurotransmission from MC is not essential for rapid pumping (Fig. 3), the E1 spikes may be a consequence of rapid pumping that do not reflect nervous system activity, leaving open the possibility that MC may act tonically.

To explore the nature of MC signaling, we examined EPGs during optogenetic stimulation of pharyngeal muscles and neurons. The MC neuron cell bodies are small (2–3 μm in diameter^16^) and are embedded in the pharyngeal muscle, which is surrounded by a thick basement membrane. As a result, electrical recording from these neurons is not currently possible. The most direct way to determine the activity pattern of these neurons would be with fluorescent calcium indicators, however GCaMP6s is not sufficiently fast to use at pumping rates near 4 Hz^57^, and we were unable to achieve adequate expression levels of the faster GCaMP6 variants.

**Figure 4:**
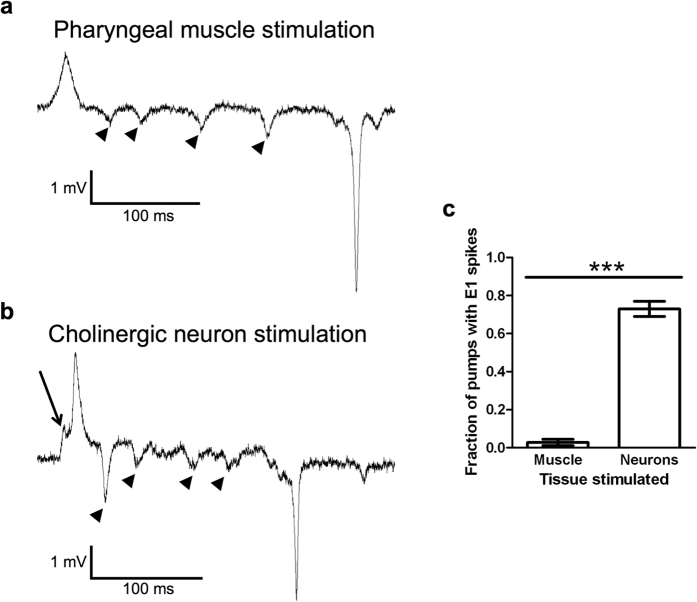
E1 spikes are detected after cholinergic neuron but not muscle stimulation. (**a**) Example EPG trace during tonic muscle stimulation. (**b**) Example EPG trace during tonic neuron stimulation. The arrow is pointing to the E1 spike, seen during tonic neuron stimulation but not tonic muscle stimulation. Arrowheads in A and B indicate P spikes due to M3 activity. (**c**) EPG recordings during optogenetic stimulation of cholinergic neurons but not pharyngeal muscle reveal E1 spikes during most pumps. We stimulated each worm 3 times for 5 seconds each time, then for each worm counted the fraction of pumps that had E1 spikes. N = 12 worms. Statistical significance was calculated using a two-tailed Student’s *t*-test. ***p < 0.001.

To determine if the E1 spike is a consequence of rapid pumping or represents an EPSP from MC, we recorded the electrical activity of the pharyngeal muscle during tonic optogenetic stimulation of the muscle in the presence of 5-HT. We found that when we stimulated the muscle to evoke rapid pumping, the E1 spike was absent from nearly all pumps (Fig. 4a,c). This result demonstrates that E1 spikes are not artifacts of rapid pumping. If MC stimulates pumping by tonic release of ACh, we would expect that tonic MC stimulation causes similar electrical activity to that seen during tonic muscle stimulation. In contrast, we found that during optogenetic stimulation of the cholinergic neurons, including the MC neurons, most pumps contained an E1 spike (Fig. 4b,c). EPG recordings during direct pharyngeal muscle stimulation contained P spikes (see arrow heads in Fig. 4a), which reflect the activity of the inhibitory glutamatergic M3 motor neurons^37^. The presence of these spikes suggests that M3 activity is directly triggered by muscle contraction in the absence of neural excitatory activity. Hence, as previously proposed^37^, M3 likely has a proprioceptive sensory function in addition to its motor neuron function.

Taken together, these results demonstrate that tonic optogenetic depolarization of the muscle stimulates pumping by increasing the rate of the myogenic rhythm, while tonic MC stimulation increases the rate of the neurogenic rhythm, suggesting that the MC neurons are rhythmically rather than tonically active (Table. 1). Thus, the pharyngeal nervous system seems to regulate pumping by a mechanism similar to that of the leech heartbeat, in which the motor neurons entrain muscle contraction rate via temporally patterned input, as opposed to the mechanism controlling the vertebrate or mollusc heartbeat, where the nervous system provides modulatory input to regulate contraction rate.

## Discussion

### A model for control of pumping rate

**Figure 5:**
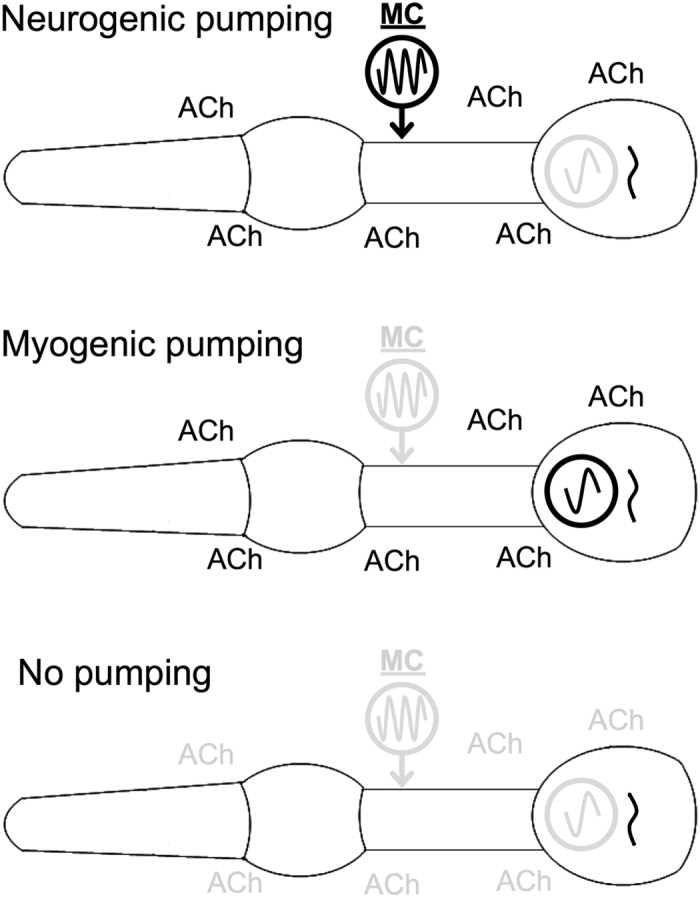
Model representing hypothesized myogenic and neurogenic mechanisms for pumping control. In intact worms, the activity of the MC neurons entrains the pharyngeal muscle and overrides the myogenic rhythm to cause rapid neurogenic pumping. When MC activity or postsynaptic response to MC activity is decreased, the myogenic rhythm sets the pumping rate. In the acute absence of nervous system function, pumping ceases completely. ACh represents acetylcholine. Circles with enclosed waveforms represent oscillators. Elements in gray are inactive.

In this work, we sought to investigate the roles of the nervous system and pharyngeal muscle in the generation of rhythmic pharyngeal pumping. Our results support a model in which the presence of tonically released ACh alters the intrinsic properties of pharyngeal muscles so that contraction and relaxation are fast enough to permit rapid and effective pumping, establishing a myogenic rhythm (Fig. 5). Alternatively, it is possible that subthreshold myogenic oscillations occur in the pharyngeal muscle, and ACh is required to allow these oscillations to produce muscle contraction. In either case, it is possible that this ACh normally comes from the pharyngeal nervous system, as the sufficiency of the extra-pharyngeal nervous system for this function is only revealed when the pharyngeal nervous system is absent. Indeed, pumping persists when the pharyngeal nervous system and muscle are dissected away from the rest of the body in the presence of 5-HT, which stimulates the MC neurons^27^,^34^.

Our results also support a model in which the cholinergic pharyngeal neurons, primarily the MC neurons, control pumping via rhythmic activity (Fig. 5). Previous reports have shown that either tonic or phasic stimulation of pharyngeal muscle is sufficient for driving rapid pumping^26^,^46^, and that tonic stimulation of pharyngeal neurons can stimulate rapid pumping^25^. Our EPGs reveal that tonic muscle stimulation induces rapid pumping but does not recapitulate the pattern of electrical activity seen during 5-HT-stimulated pumping, which requires the MC neurons^27^. This result suggests that tonic muscle stimulation increases the rate of the myogenic rhythm rather than mimicking neural activity.

Our EPG recordings indicate that although MC is not active during pumping induced by optogenetic muscle stimulation, M3 is active (Fig. 4a). Taken together with previous work, this finding suggests that M3 fires action potentials in response to pharyngeal muscles contraction^37^. The resulting IPSPs shorten the duration of muscle contraction and contribute to effective food transport^37^,^42^,^48^. This proprioceptive feedback demonstrates an additional layer of complexity in the pharyngeal circuit as it shows that some neurons can have both sensory and motor functions, a phenomenon previously described in a class of motor neurons that regulate *C. elegans* locomotion^58^. In the future, the combination of genetic manipulations and the monitoring of neuron and muscle activity with genetically encoded calcium or voltage sensors will provide more information about the activity of pharyngeal nervous system, permitting a better understanding of the function of this network^59^.

### Why does the pharynx require the nervous system for pumping?

Muscular pacemakers such as the vertebrate, leech, and mollusc hearts contract rhythmically when isolated from their respective nervous systems, but the pharyngeal muscle requires an extrinsic factor to exhibit pumping. While the pharynx and vertebrate heart have many similar ionic conductances, one striking difference between them is that the *C. elegans* genome does not have any homologs of genes encoding HCN channels, which mediate the hyperpolarization-activated mixed cation current in the vertebrate heart referred to as the pacemaker current^60^. The absence of the so-called pacemaker current in the pharynx could explain why the pharynx does not spontaneously contract in the absence of extrinsic factors, as the membrane may not depolarize in response to hyperpolarization.

Recent evidence suggests that in addition to the well-characterized membrane oscillator in the vertebrate heart, a Ca^2+^ oscillator may act simultaneously and work with the membrane oscillator to mediate contraction^2^. The ryanodine receptor is critical for vertebrate heart function, but the gene encoding the *C. elegans* ryanodine receptor (*unc-68*) is relatively unimportant for pharyngeal function^61^, and there is no evidence of a corresponding Ca^2+^ oscillator in the pharynx. Additionally, while the K^+^ channel responsible for pharyngeal repolarization (EXP-2) has generally similar properties to the vertebrate HERG channel, it is structurally dissimilar^50^, suggesting that it may have subtle differences in function that affect the stability of the muscle membrane potential and its ability to generate rhythmic activity.

### Multiple mechanisms for modulation of pumping rate

The results described here have implications for understanding how neuromodulators influence pumping rate. Many neuromodulators, including biogenic amines^62^ and neuropeptides^63^, are released from pharyngeal and extra-pharyngeal neurons^64^,^65^ and a subset of these stimulate or inhibit pumping. Multiple lines of evidence suggest that these modulators may act on either the pharyngeal neurons or muscles^46^,^63^. For example, the extra-pharyngeal nervous system could non-synaptically regulate pump rate by altering the myogenic rhythm, bypassing the pharyngeal nervous system, in a mechanism similar to that of vertebrate hearts, or by altering the neurogenic rhythm. The identification of two distinct pacemakers capable of producing rapid pumping suggests that there are many different mechanisms by which neuromodulators can influence pharyngeal pumping, even if their effects on behavior are similar^25^,^46^.

## Materials and Methods

### Worm strains and cultivation

We performed all experiments with adult hermaphrodites. Unless otherwise specified, animals were cultivated on the surface of NGM agar in a 20 °C incubator. Strains used include **YX11** *vsIs48*[*Punc-17::GFP*] *X; zxIs6*[*Punc-17::ChR2(H134R)::YFP; lin-15*(+)]^25^, **CX16557** *kyIs5640*[*Pmyo-2::Chrimson; Pelt-2::his4.4-mCherry*]^46^, **YX87** *eat-2(ad1113) II; kyIs5640*[*Pmyo-2::Chrimson; Pelt-2::his4.4-mCherry*], **YX97** *unc-18(e81) X; kyIs5640*[*Pmyo-2::Chrimson; Pelt-2::his4.4-mCherry*], **CX14373** *kyEx4571*[*Ptag-168::HisCl1::SL2::GFP; Pmyo-3::mCherry*]^38^, and **YX96** *kyIs5640*[*Pmyo-2::Chrimson; Pelt-2::his4.4-mCherry*]*; kyEx4571*[*Ptag-168::HisCl1::SL2::GFP; Pmyo-3::mCherry*].

### Optogenetics

We performed optogenetic stimulation of pharyngeal neurons and muscles as previously described^25^,^46^,^66^. All experiments were performed on 10% agarose pads containing 10 mM serotonin (5-HT) mounted on glass slides, except experiments involving histamine, which were performed on a 2% agarose pad lacking 5-HT, and electrophysiological recordings, which were performed in Dent’s saline^67^ with 10 mM 5-HT. For experiments using weak optogenetic stimulation, we used a red LED with an irradiance of approximately 0.35 mW/mm^2^. For strong optogenetic stimulation, we used ~37 mW/mm^2^, as described previously^25^,^46^,^66^.

### High speed imaging

We performed all high speed imaging experiments with the strain **YX96** *kyIs5640*[*Pmyo-2::Chrimson; Pelt-2::his4.4-mCherry*]*; kyEx4571*[*Ptag-168::HisCl1::SL2::GFP; Pmyo-3::mCherry*] on 2% agarose pads in the presence of OP50 bacterial food. For control and experimental worms, we imaged pharyngeal behavior using an infrared LED (800–850 nm wavelength) placed directly above the condenser of a Leica DMI 3000B inverted microscope. This wavelength was selected to avoid stimulating Chrimson. We recorded behavior at 500 frames per second using a Vision Research Phantom v9.1 CMOS camera, then manually annotated pumping events with the help of custom MATLAB scripts. For the experimental group, we added 10 mM histamine to the agarose pads. We stimulated pumps using 200 ms pulses of the same red LED used for other weak optogenetic experiments. Since the pan-neuronal HisCl-containing transgene was present as an extrachromosomal array, expression patterns were variable between worms. We therefore reasoned that the worms with the most abnormal behavior were those with the broadest expression, and thus only analyzed recordings for worms in which the behavior was most abnormal, those in which 200 ms stimulation caused only a single pump. Subsequent experiments after integrating this transgene produced qualitatively similar results.

### Electropharyngeograms

EPGs were performed as previously described^37^,^67^. We performed EPGs on intact first day adult worms. We created recording chamber by making a ring of vacuum grease on a cover slide, then filled the chamber with Dent’s saline^67^ containing 10 mM serotonin. 10–15 worms were added to the chamber for each experiment. Worms were then sucked into glass microelectrodes, which were connected by a silver chloride coated silver wire to an Axon Instruments CV-7B headstage. The headstage was connected to Axon Instruments MultiClamp 700B amplifier and DigiData 1440A digitizer. Electrodes were fabricated on a Sutter P-1000 micropipette puller using borosilicate glass with an inner diameter of 0.5 mm. Electrodes were pulled to an inner diameter of approximately 20 μm. We performed optogenetic stimulation^25^,^46^,^66^, using 5 s light pulses separated by 5 s, with the exception of the experiments with worms expressing the pan-neuronal HisCl channel, where we used 200 ms light pulses. All recordings were performed in current clamp mode. E1 spikes were identified by manual observation of the EPG traces using the Axon Instruments pCLAMP 10 software package.

## Additional Information

**How to cite this article**: Trojanowski, N. F. *et al.* Pharyngeal pumping in *Caenorhabditis elegans* depends on tonic and phasic signaling from the nervous system. *Sci. Rep.* **6**, 22940; doi: 10.1038/srep22940 (2016).

## Figures and Tables

**Table 1:** Summary of key results in this paper and other relevant results^25^,^27^,^46^.

	Presence of 5-HT				Tonic neuron stimulation				Tonic muscle stimulation			
	NS intact		NS defective		NS intact		NS defective		NS intact		NS defective	
Pump rate	Rapid	Ref. 27	Slow	Ref. 27	Rapid	Ref. 25	Slow	Ref. 25	Rapid	Ref. 46	Rapid	Fig. 3
E1 spikes	Yes	Ref. 27	No	Ref. 27	Yes	Fig. 4B	Not Tested		No	Fig. 4A	Not Tested	

References or figures for each observation are listed in the table. Tonic optogenetic stimulation of pharyngeal neurons causes rapid pumping with E1 spikes, representing activation of the neurogenic rhythm and mimicking rapid pumping in the presence of food. In contrast, tonic optogenetic stimulation of pharyngeal muscle causes rapid pumping without E1 spikes, representing activation of the myogenic rhythm and mimicking pumping seen in mutants with defective (but not abolished) rhythmic nervous system input onto pharyngeal muscle, such as eat-2 mutants. In these mutants, activation of the myogenic but not neurogenic rhythm increases pumping rate, as expected.
